# Spatial transcriptomics reveals that metabolic characteristics define the tumor immunosuppression microenvironment via iCAF transformation in oral squamous cell carcinoma

**DOI:** 10.1038/s41368-023-00267-8

**Published:** 2024-01-30

**Authors:** Zheqi Liu, Zhen Zhang, Yu Zhang, Wenkai Zhou, Xu Zhang, Canbang Peng, Tong Ji, Xin Zou, Zhiyuan Zhang, Zhenhu Ren

**Affiliations:** 1 Department of Oral and Maxillofacial - Head and Neck Oncology, Shanghai Ninth People’s Hospital, Shanghai Jiao Tong University School of Medicine; College of Stomatology, Shanghai Jiao Tong University; National Center for Stomatology; National Clinical Research Center for Oral Diseases; Shanghai Key Laboratory of Stomatology & Shanghai Research Institute of Stomatology, Shanghai, China; 2 Department of Oral and Maxillofacial Surgery, Zhongshan Hospital, Fudan University, Shanghai, China; 3 School and Hospital of Stomatology, Kunming Medical University, Kunming, China; 4 Center for Tumor Diagnosis & Therapy, Jinshan Hospital, Fudan University, Shanghai, China; 5 Department of Pathology, Jinshan Hospital, Fudan University, Shanghai, China; 6 Hainan Western Central Hospital, Academician Zhang Zhiyuan Team Innovation Center, Danzhou, China

**Keywords:** Cancer metabolism, Oral cancer

## Abstract

Tumor progression is closely related to tumor tissue metabolism and reshaping of the microenvironment. Oral squamous cell carcinoma (OSCC), a representative hypoxic tumor, has a heterogeneous internal metabolic environment. To clarify the relationship between different metabolic regions and the tumor immune microenvironment (TME) in OSCC, Single cell (SC) and spatial transcriptomics (ST) sequencing of OSCC tissues were performed. The proportion of TME in the ST data was obtained through SPOTlight deconvolution using SC and GSE103322 data. The metabolic activity of each spot was calculated using scMetabolism, and k-means clustering was used to classify all spots into hyper-, normal-, or hypometabolic regions. CD4T cell infiltration and TGF-β expression is higher in the hypermetabolic regions than in the others. Through CellPhoneDB and NicheNet cell-cell communication analysis, it was found that in the hypermetabolic region, fibroblasts can utilize the lactate produced by glycolysis of epithelial cells to transform into inflammatory cancer-associated fibroblasts (iCAFs), and the increased expression of HIF1A in iCAFs promotes the transcriptional expression of CXCL12. The secretion of CXCL12 recruits regulatory T cells (Tregs), leading to Treg infiltration and increased TGF-β secretion in the microenvironment and promotes the formation of a tumor immunosuppressive microenvironment. This study delineates the coordinate work axis of epithelial cells-iCAFs-Tregs in OSCC using SC, ST and TCGA bulk data, and highlights potential targets for therapy.

## Introduction

Oral squamous cell carcinoma (OSCC) is the most common malignant tumor of the head and neck region, accounting for more than 90% of head and neck cancer.^1,2^ Patients usually experience difficulties in chewing, speaking, and breathing after surgery, and the 5-year survival rate is low in late-stage patients. Risk factors for OSCC include tobacco and/or alcohol consumption, areca chewing, and unhealthy oral hygiene, which lead to chronic local inflammation caused by physical, chemical, or biological factors. Although studies have revealed a series of molecular events associated with the disease, a comprehensive understanding of the spatial characteristics and interactions within the tumor microenvironment (TME) is still lacking.

Carcinogenesis relies on a series of events involved in cell metabolism reprogramming and TME reshaping. Cancers in the head and neck regions exhibit significant hypoxic features^3^ resulting from microvascular malformation and rapid proliferation. Single-cell transcriptomics has shown the TME subclusters in detail^4,5^ and explained their roles in altering the immune status.^6,7^ However, the association between metabolic activity and TME conversion (including function and recruitment/infiltration) remains unclear. It is suggested that hypoxia-driven metabolism reprogramming would lead to tumor immune escape, with the malfunction of pro-inflammatory factors and stabilization of co-inhibitory factors. As a result, the tumor microenvironment is spatially distinct based on their hypoxia-related metabolic activities, the compositions and inter-cell communications are supposed to be differed due to the metabolic background of the regions.

In the current study, the spatial transcriptomic (ST) technique of the 10× Visium platform was applied to three OSCC samples and their adjacent normal tissues, cell type proportions were computed with both our own and publicly available OSCC single-cell sequencing data by Spotlight. By distinguishing the spatial clusters using their metabolic status, it was observed that regions under different metabolic status exhibits distinct cell composition and cell-cell communications. A complex trajectory involving cancer cells, cancer-associated fibroblasts (CAFs), and regulatory T cells (Tregs) has been identified. Through bioinformatics and correlation analyses, we found that the transcription factors, ligands, receptors, and target genes involved in this process synergistically to promote immune suppression and cancer progression. These findings highlight potential targets for combined therapy with immune checkpoint blockade (ICB) to improve treatment outcomes. In summary, our study has explored the spatial relationship between cancer regional metabolism and the TME in OSCC. Our results shed light on the underlying mechanisms of OSCC development and have important implications for clinical practice, particularly in the treatment of this disease.

## Results

### Spatial clustering of oral squamous cell carcinoma tissue samples

**Fig. 1:**
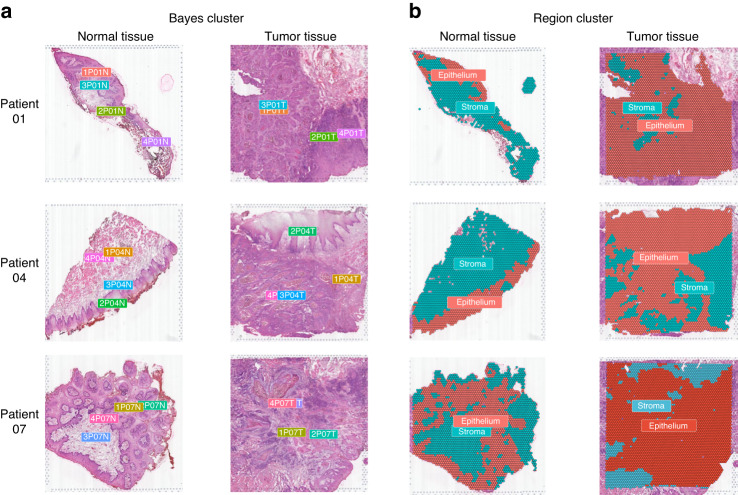
Spatial clustering of oral squamous cell carcinoma tissue samples. **a** Spatial clustering of BayesSpace was used to identify distinct regions within the tissue samples, and each sample was analyzed independently. **b** Based on the Bayes clusters, unsupervised clustering was used to divide the tissue regions into epithelial and stromal regions, which was confirmed by histological morphological information from hematoxylin & eosin (H&E) staining slides

After quality control screening, 20,195 tissue spots of six samples from three patients were sequenced (Table S1). Integrated clustering analysis of ST data from all tissue spots revealed seven distinct clusters (Fig. S1A). Spatial clustering analysis was performed, and each tissue sample was divided into four spatial clusters using the BayesSpace package^8^ (Fig. 1a). Based on the results of spatial clustering, expression characteristics of unsupervised clustering (Fig. S1C), and histological morphological information of hematoxylin and eosin (H&E)-stained slides (Fig. S1D), we divided tissues into epithelial and stromal regions (Fig. 1b). The distribution of epithelial and stromal spots in tumor and normal tissues is shown in Fig. S1B.

### Metabolic heterogeneity affects the TME

**Fig. 2:**
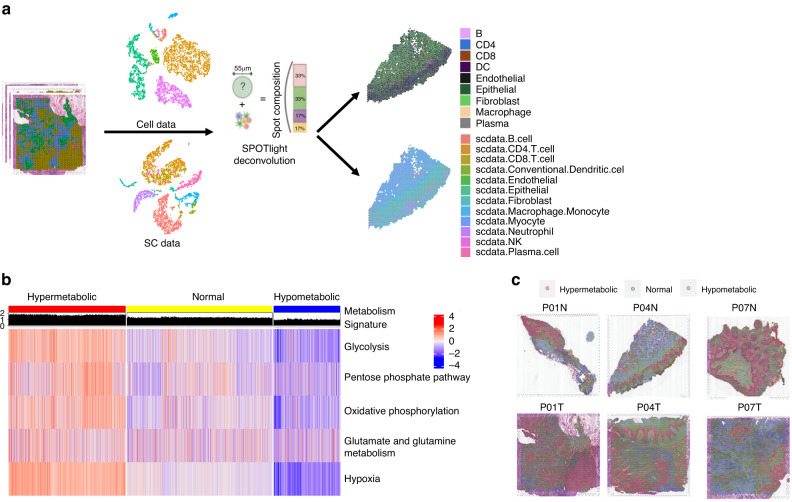
Metabolic heterogeneity of the oral squamous cell carcinoma tissue samples. **a** The SPOTlight deconvolution method was used to calculate the proportion of various cell types in each spot, using Cell data and SC data as reference. **b** Heatmap shows the metabolic score representing glycolysis, pentose phosphate pathway, oxidative phosphorylation, glutamate/glutamine metabolism, and hypoxia for each spot. All spots were categorized into three clusters based on their metabolic activity: hypermetabolic, normal-metabolic, and hypometabolic. **c** Representative spatial transcriptomic tissue slides show the spatially projected metabolism clusters

**Fig. 3:**
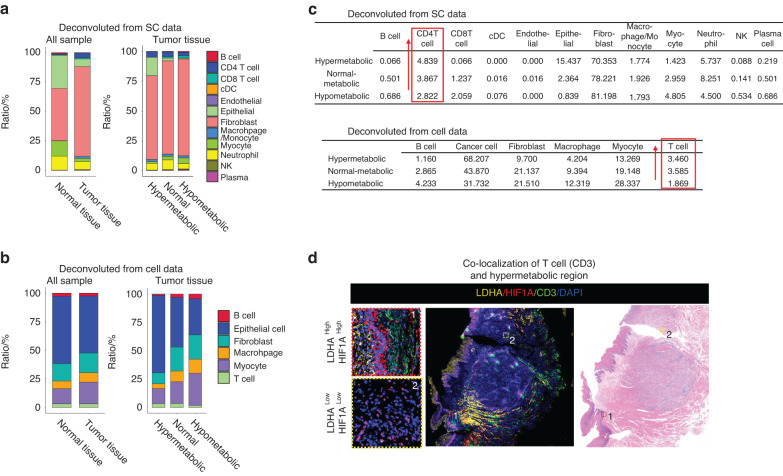
Metabolic heterogeneity affects the TME. **a** Deconvolution results from Cell data indicate the proportion of each cell type in different types of tissue and metabolic regions of tumors. **b** Deconvolution results from SC data indicate the proportion of representative cell type of each spot in different types of tissue and metabolic regions of tumors. **c** The detailed percentage of different cell types in Cell data and SC data is shown, with T cells and CD4T cells displaying a similar changing trend in different metabolic regions. **d** Immunofluorescence shows the co-localization of T cells (CD3) and hypermetabolic regions (LDHA and HIF1α) in OSCC

To analyze the proportion of cells in each spot in the spatial dataset, we utilized two sets of single-cell data: one set of single-cell sequencing data, named SC data, obtained from clinical oral squamous cell carcinoma tissue samples using the 10X Genomics platform, and another set of OSCC single-cell data from Puram, S. V, et al. (GSE103322,^4^) named Cell data. Our single-cell data underwent processing with Seurat’s standard single-cell pipeline, and cell-type labeling was performed with various cell markers (Fig. S2A). Subsequently, we used the SPOTlight^9^ algorithm to deconvolute the spatial groups’ spots with the two sets of single-cell data as a reference to obtain the proportion of cells in each spot (Fig. 2a, Supplementary Materials). To evaluate the metabolic activity of each spot, we selected five metabolism-related pathways, including glycolysis, pentose phosphate, oxidative phosphorylation, glutamate/glutamine metabolism, and hypoxia, to explore the metabolic status of the OSCC samples. Using the scMetabolism^10^ we calculated the metabolic scores of the five pathways and reflected them onto tissue slides (Fig. S3A–E). Furthermore, we calculated the mean metabolic scores of the five pathways as the metabolic signature, which represents the metabolic activity of each spot in the tissues (Fig. S3F). The total spots were clustered into three categories: hypermetabolic, normal metabolic, and hypometabolic (Fig. 2b). By mapping the three clusters onto tissue slides and comparing the results of spatial clustering, we found that the hypermetabolic region was in the epithelial region (Fig. 2c), which was consistent with the hypermetabolic state of epithelial cells in tumor tissues. The cell type with the highest proportion in each spot was set as the main cell type, and the proportion of different cell types in different metabolic regions was computed. By calculating the proportion of different cell types, we found that the proportion of T cells (Cell data) and CD4 T cells (SC data) increased with the boosting of metabolic activity (Fig. 3a–c). We also performed immunofluorescence (IF) and found that the proportion of T cells (CD3) was dominate in regions with over-expressed LDHA and HIF1α (Fig. 3d). These results suggest that changes in metabolic activity status in tumor tissues affect the composition of the TME in OSCC, particularly the proportion of CD4 T cells.

### Tregs are enriched in the hypermetabolic regions

**Fig. 4:**
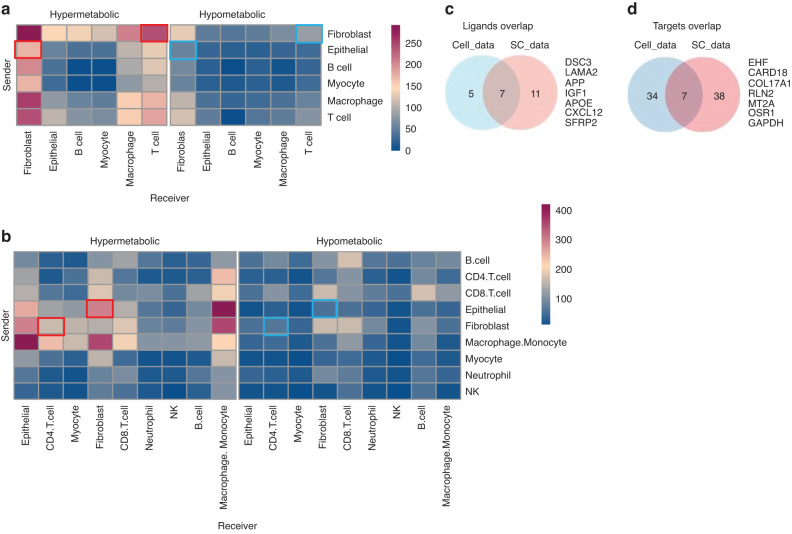
Cell communications in different metabolic regions of tumor tissues. Heatmap showing the counts of cell-cell communication between different cell types in the three metabolic regions. **a** Cell data deconvolution and **b** SC data deconvolution. The red and blue boxes indicate the communication between fibroblast-T (CD4 T) and Epithelial-fibroblast in hypermetabolic and hypometabolic regions, respectively. The blue box indicates changes in the communication of fibroblasts. **c** Intersection of predicted ligands from Cell data and SC data through Nichenet analysis. **d** Intersection of predicted target genes for the predicted ligands from Cell data and SC data through Nichenet analysis. The 7 target genes were used as the target panel below

Cell-cell communication is the primary mechanism of interaction between different cells in the TME. We used CellPhoneDB^11^ to analyze cell-cell interactions in different metabolic regions of the tumor tissue. Compared to hypometabolic regions, we found that the communication counts between fibroblasts and CD4 T cells, and between epithelial and fibroblast cells in hypermetabolic regions were significantly higher (Fig. 4a, b, red and blue boxes). Since we observed a change in the proportion of CD4 T cells in hypermetabolic regions, we focused on studying the CD4 T cells first.

NicheNet^12^ was adopted to evaluate ligand activity from sender cells (fibroblasts) and potential target genes in CD4 T cells. We found that 12 and 18 top-ranked ligands were predicted in Cell data SC data, respectively (Table S2). After taking the intersection of the ligands list, 7 overlapped ligands were identified (Fig. 4c). We then extracted the matrix of ligands-targets genes of T cells (CD4 T cells) in Cell data and SC data (Tables S3 and S4) and performed the intersection operation to obtain 7 overlapped target genes (Fig. 4d).

**Fig. 5:**
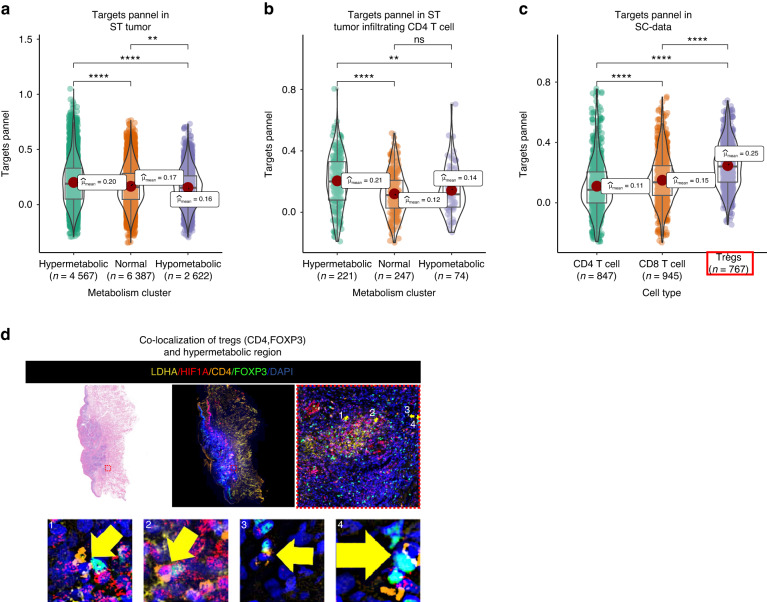
Tregs accumulate in the hypermetabolic regions. **a** Expression of the target panel in different metabolic regions of spatial data (ST data). **b** Expression of the target panel in different metabolic regions of tumor-infiltrating CD4 T cells. **c** Expression of the target panel in CD4, CD8, and Tregs of SC data. The target panel has the highest expression in Tregs (red box). **d** Immunofluorescence shows the co-localization of Tregs (CD4, FOXP3) and hypermetabolic regions (LDHA and HIF1A) in OSCC. Yellow arrows indicates the cells that have co-expression of FOXP3 (Green) and CD4 (Orange). *P < 0.05, **P < 0.01, ***P < 0.001, ***P < 0.000 1

With the AddModuleScore function of Seurat, we mapped this 7 genes panel onto ST data. The results showed that in tumor samples and tumor-infiltrating CD4 T cells, these genes were significantly over-expressed in hypermetabolic regions (Fig. 5a, b). To determine the precise T cell lineage associated with these genes, we mapped it onto the SC data and found that the panel was highly expressed in regulatory T cells (Tregs) (Fig. 5c). Immunofluorescence (IF) also confirmed the colocalization of Tregs (CD4, FOXP3) and high metabolic regions (LDHA ^High^, HIF1A ^High^) (Fig. 5d).

### Fibroblast derived CXCL12 recruits Tregs to hypermetabolic regions

**Fig. 6:**
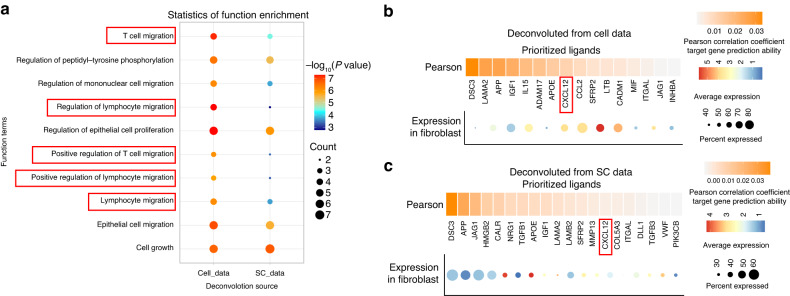
Fibroblast derived CXCL12 recruit Tregs to hypermetabolic regions. **a** The top 10 biological process functional items predicted by Nichenet analysis for fibroblast ligands include several related to T or lymphocyte migration (highlighted in red box). **b**, **c** Show the expression and ligand activity prediction of prioritized ligands for fibroblast using Nichenet analysis. Panel (**b**) displays data from Cell data and panel (**c**) displays data from SC data

**Fig. 7:**
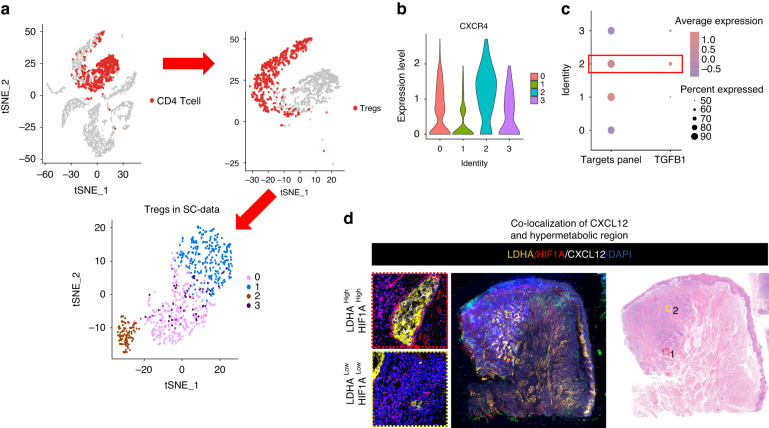
Heterogeneity of Tregs. **a** Tregs were isolated from CD4 cells in single-cell data and subjected to unsupervised clustering, resulting in four distinct groups. **b** A violin plot depicts the expression of CXCR4 in the four Tregs clusters. Cluster 2 exhibits the highest expression of CXCR4. **c** A dotplot illustrates the expression of the targets panel and TGFB1 in the four Tregs clusters. Cluster 2 displays the highest expression of both. **d** Immunofluorescence staining shows the co-localization of CXCL12 and hypermetabolic regions (LDHA and HIF1α) in OSCC

Cell infiltration in tumors is mainly driven by chemokines. We performed functional enrichment analysis of predicted ligands and found that several biological processes related to T cell or lymphocyte migration were among the top 10 enriched items (Fig. 6a, red box). CXCL12 was a common chemokine found in both datasets (Fig. 6b, c). Tregs in the SC data were further divided into 4 clusters (Fig. 7a). Although the main receptors of CXCL12 are ACKR3 and CXCR4, ACKR3 had no expression in Tregs according to the single-cell analysis (Fig. S4A), so we focused on CXCR4 expression. Cluster 2 of Tregs had the highest expression of CXCR4 and the 7-gene target panel identified from the ST data (Figs. 3d & 7b). Tregs are known to secrete TGF-β, which creates an immunosuppressive environment in tumors. We examined the expression of cytokines secreted by T cells and found that only TGF-β (TGFB1) expression increased in both hypermetabolic and hypometabolic regions (Fig. S4B, S4C). In addition, TGFB1 had the highest expression in Cluster 2 of Tregs in the SC data (Fig. 7c). Finally, we confirmed the co-localization of CXCL12 and high metabolic regions (LDHA ^High^, HIF1α ^High^) through immunofluorescence, supporting the conclusion that a cluster of Tregs is recruited by CXCL12 in the hypermetabolic regions in OSCC (Fig. 7d).

### Cancer-derived lactate induces iCAFs to express CXCL12

The up-stream cell communication is the talks between epithelial cells and fibroblasts (Fig. 4a, b). Based on the metabolism activities of OSCC hypermetabolic region, cancer cells mainly obtain energy through aerobic glycolysis (known as the Warburg effect), producing lactic acid as the main metabolite. Immune and stromal cells in the TME uptake lactate for energy production.^13–15^ Previous studies have suggested that inflammatory cancer-associated fibroblasts (iCAFs) have relatively high expression of pyruvate carboxylase genes^13^ and can regulate the TME by secreting cytokines such as CXCL12,^16,17^ which was shown to be an important chemokine in recruiting Tregs in our previous results.

To conduct in vitro cell experiments, we cultured CAF cells and cancer cells (HN6 cell line) with the same number of cells under the environment of high and low glucose culture medium, and detected the content of lactate secreted in the supernatant. The results suggested that lactate secretion of HN6 cells increased significantly in the high-glucose environment, and lactate content was decreased in the supernatant of CAFs (Fig. S5A).These results suggest that cancer cells can produce a large amount of lactate under high glucose environment, while CAF cells are lactic acid consuming cells and lack the ability of lactate production.

**Fig. 8:**
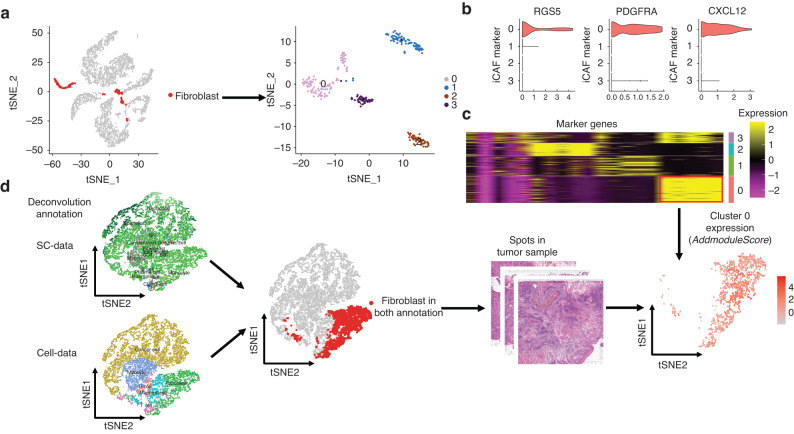
Secretion of CXCL12 is mainly from iCAF. **a** Fibroblasts were extracted from SC data and clustered into four groups using unsupervised clustering. **b** Violin plot showing the expression of RGS5, PDGFRA, and CXCL12 in the four fibroblast clusters. Cluster 0 exhibited the highest expression of all three markers. **c** Heatmap displaying the expression of specific marker genes for the four fibroblast clusters. The marker genes of Cluster 0 were chosen for further analysis. **d** Analysis of spots that were identified as fibroblasts in both the SC data and spatial transcriptomics (ST) data. The marker genes of Cluster 0 were mapped onto these spots

**Fig. 9:**
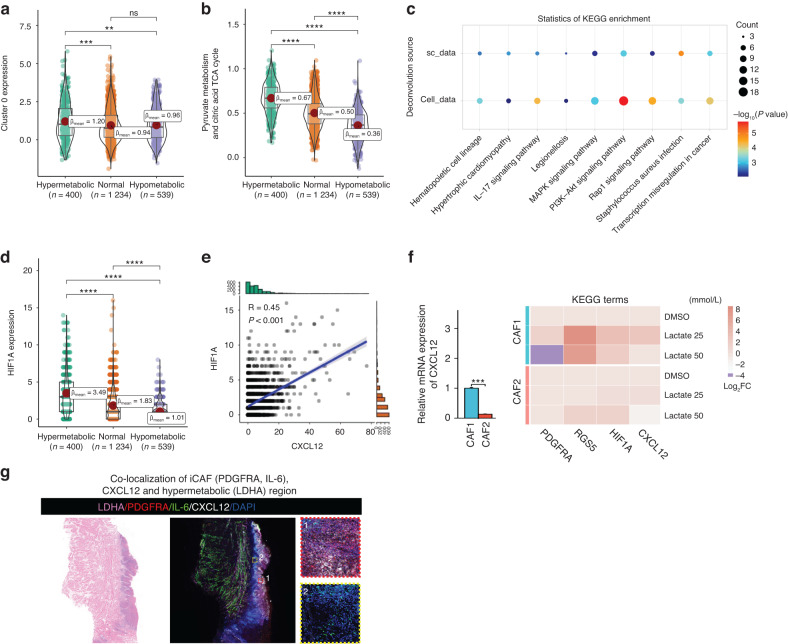
Lactate promotes the transition of fibroblasts to iCAF. **a** Violin plot showing the expression of Cluster 0 in different metabolic regions in the ST data. Expression in hypermetabolic regions was higher than that in hypometabolic regions. **b** Metabolic activity scores of pyruvate metabolism and citric acid cycle in fibroblasts in different metabolic regions. Pyruvate metabolic activity increased gradually with increasing metabolic activity. **c** Top 10 KEGG functional pathways of predicted target genes of fibroblasts through Nichenet analysis. **d** HIF1A expression in tumor-infiltrating fibroblast spots in different metabolic regions. HIF1A expression was higher in hypermetabolic regions. **e** Correlation scatter plot of HIF1A expression and CXCL12 expression in tumor-infiltrating fibroblast spots. **f** RT-qPCR analysis of iCAF markers (PDGFRA, RGS5), HIF1A, and CXCL12 expression in 2 CAF cell lines after 72 h of treatment with lactate. **g** Immunofluorescence staining shows the co-localization of iCAF (PDGFRA and IL-6), CXCL12 and hypermetabolic regions (LDHA) in OSCC

We extracted fibroblast data from the SC data and divided it into 4 clusters (Fig. 8a). Cluster 0 had the highest expression of CXCL12 and iCAF markers (PDGFRA and RGS5) among the 4 clusters (Fig. 8b). We then identified the specific marker genes for Cluster 0 (Fig. 8c) and mapped these genes to ST spots defined as fibroblast in both the Cell data and SC data (Fig. 8d). The expression of the Cluster 0 panel was consistent with the metabolic activity in ST data (Fig. 9a).

We also analyzed the metabolic activity of pyruvate metabolism and the citric acid cycle in our ST data using the scMetabolism. The results showed that pyruvate metabolism activity gradually increased with an increase in overall metabolic activity (Fig. 9b). Additionally, the monocarboxylate transporter MCT1 (SLC16A1), which plays a key role in lactate transport, was highly expressed in the hypermetabolic regions (Fig. S5B).

Similarly, we extracted target genes corresponding to overlapped ligands from epithelial cells in both datasets using the Nichenet results, and performed KEGG functional enrichment analysis of these target genes. The results showed that multiple signaling pathways were activated in fibroblast that were communicating with epithelial cells (Fig. 9c). Previous studies have shown that the PI3K-Akt pathway can regulate HIF1A and further regulate the transcriptional expression of downstream genes.^18–20^ HIF1A has also been confirmed to be an upstream transcriptional regulator of CXCL12.^21,22^ We analyzed the expression of tumor-infiltrating fibroblasts in the ST data and found that HIF1A expression was increased in fibroblasts in hypermetabolic regions (Fig. 9d), and there was a significant correlation between HIF1A and CXCL12 expression (Fig. 9e).

To validate these findings, we performed in vitro experiments. After primary culture of 2 CAF cell lines (CAF1 and CAF2)from OSCC patients, sodium lactate was added to cells, and gene expression was measured after 72 h. The expression of CXCL12 in CAF1 was much higher than that in CAF2, and that the expression of iCAF markers (PDGFRA, RGS5), HIF1A and CXCL12 were up-regulated after lactate treatment. The increase of these marker in CAF1 was larger than CAF2 according to the fold change value (Fig. 9f). Immunofluorescence staining shows the co-localization of iCAF (PDGFRA and IL-6),CXCL12 and hypermetabolic regions (LDHA) in OSCC (Fig. 9g).

In summary, lactate secreted by cancer cells can promote the transformation of fibroblasts into iCAFs and the expression of CXCL12, possibly via the PI3K-Akt-HIF1A axis.

### Cancer cell-iCAF-Treg recruitment in hypermetabolic OSCC

**Fig. 10:**
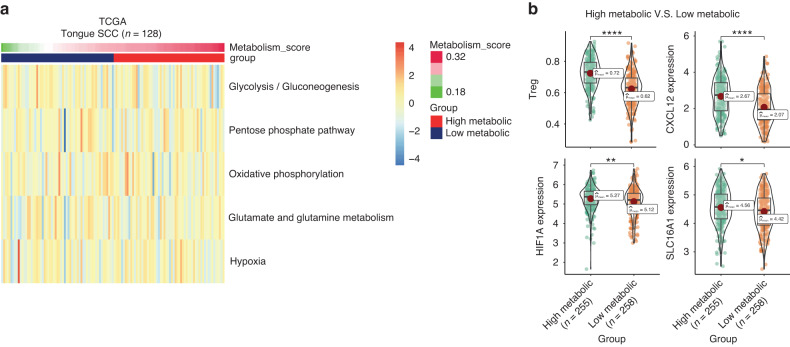
Validation of the OSCC hypermetabolic signature. **a** This heatmap displays the metabolic score of five pathways (glycolysis, pentose phosphate pathway, oxidative phosphorylation, glutamate/glutamine metabolism, and hypoxia) in OSCC samples from The Cancer Genome Atlas. Samples were divided into two clusters based on the median score of their metabolic signature. **b** The expression of Treg infiltration, CXCL12, HIF1A, and SLC16A1 was found to be upregulated in samples with high metabolic scores compared to those with low metabolic scores

**Fig. 11:**
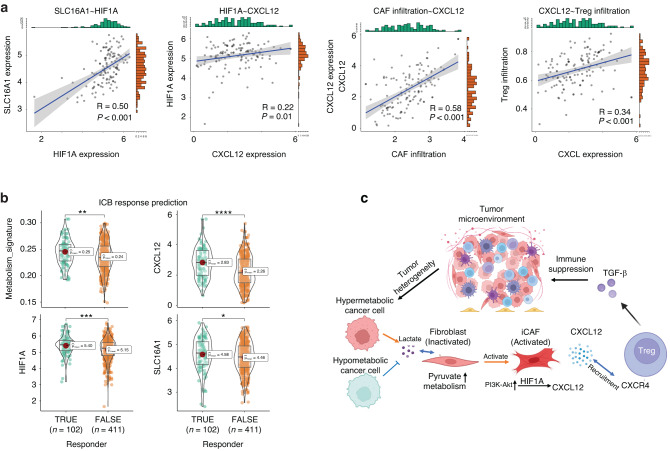
Cancer cell-inflammatory cancer-associated fibroblasts (iCAFs)-regulatory T cells (Treg) recruitment in hypermetabolic oral squamous cell carcinoma (OSCC). **a** This correlation scatter plot shows a strong positive correlation between SLC16A1 and HIF1A, HIF1A and CXCL12, CAF infiltration and CXCL12, and CXCL12 and Treg infiltration in OSCC samples. **b** The TIDE algorithm predicted that immune checkpoint blockade-responsive OSCC tumor samples had higher scores for metabolic signature, CXCL12, HIF1A, and SLC16A1 expression compared to non-responsive samples. **c** In hypermetabolic OSCC samples, lactate utilization (SLC16A1), iCAF, HIF1A, CXCL12, and Treg chemotaxis contribute to immune suppression in the tumor microenvironment. *P < 0.05, **P < 0.01, ***P < 0.001, ***P < 0.000 1

We utilized RNA-seq data from The Cancer Genome Atlas (TCGA) to validate our ST results for OSCC. Using the scMetabolism algorithm, we assessed the metabolic pathway scores of 128 OSCC samples and calculated the mean of five pathway scores to obtain the metabolism score, as in the ST data. The median metabolism score was used as the cutoff value to classify all samples into high and low metabolic groups (Fig. 10a). Using gene sets from the Bindea study,^23^ we calculated the immune cell infiltration of each sample by ssGSEA, and the infiltration of CAF was calculated using MCPCounter (Fig. S5C). By comparing the high- and low-metabolism groups, we found that the proportion of infiltrating Tregs in the high-metabolic group was significantly higher than that in the low-metabolic group. The key molecules identified in the ST data analysis, such as CXCL12, HIF1A, and SLC16A1, were also significantly upregulated in the high-metabolic samples (Fig. 10b). iCAF markers RGS5 and PDGFRA also showed higher expression in the high metabolic samples (Fig. S5D). Correlation analysis further revealed strong positive correlations between SLC16A1 and HIF1A, HIF1A and CXCL12, CAF infiltration and CXCL12, CXCL12 and Treg infiltration (Fig. 11a). The above results suggest a biological positive correlation between enhanced lactate utilization and increased expression of HIF1A, CXCL12, and CAF infiltration, as well as increased Treg infiltration. The iCAF markers RGS5 and PDGFRA also showed strong positive correlation with HIF1A and CXCL12 in OSCC samples (Fig. S5E). Additionally, we used TIDE^24,25^ to estimate the response of TCGA oral cancer samples to immune checkpoint blockade (ICB) therapy (Supplementary materials). Comparing the results showed that the metabolism scores of ICB-responder samples were significantly increased, and correspondingly, the expression of CXCL12, HIF1A, and SLC16A1 was higher than that in non-responder samples (Fig. 11b). These findings suggest the existence of a phenomenon of lactate utilization (SLC16A1)/iCAF/HIF1A/CXCL12/Treg chemotaxis in hypermetabolic samples in OSCC, resulting in immune suppression in the TME (Fig. 11c).

## Discussion

Low levels of molecular oxygen are frequently observed in primary tumor subregions, with the highest hypoxia scores found in squamous cell tumors, particularly in the case of HNSCC.^3^ An anoxic status leads to harsh metabolic and physical microenvironments, including imbalanced regulation of cancer cells, fibroblasts, and various immune cells. A tumorigenic cascade is mediated by HIF-1α-related signaling, which leads to a microenvironment beneficial for glycolysis and lactic acid production.^26^ However, there is a lack of studies on the relationship between regional metabolic heterogeneity and corresponding immune cell activities. Via the application of spatial transcriptomic technology and downstream data analysis, the TME of oral cancer tissue spots were distinguished by hypoxia metabolic scores. The hypermetabolic regions were dominated by epithelial tissues, and the composition of immune cells, including the ratio of T cells, B cells, and macrophages, varied significantly.

Based on the findings of inter-cell communication, predicted target gene enrichment, and differential expression of cytokines, it was discovered that TGF-β was over-secreted in hypermetabolic regions by Tregs. Furthermore, the proportion of TGF-β-secreting T cells was found to be significantly increased in these areas. It is widely acknowledged that the elimination of lactic acid by cancer cells during glycolysis is a primary inducer of tumor-infiltrating Tregs,^27^ which strongly suppress antitumor cytotoxicity.^28^ The current study proposes that Treg infiltration in OSCC is not merely regulated by lactic acid but also by chemotaxis, which was also validated by the Multiple fluorescence staining.

Our results indicate that fibroblasts play a crucial role in recruiting Tregs to hypermetabolic regions through the interaction of CXCL12-ACKR3.CXCL12 upregulation has been reported to be one of the representative events of iCAF,^22^ which was validated by our data. Fibroblasts require lactic acid to promote the transformation to CAF,^13^ the overexpression of MCT1, activation of PI3K-AKT, and MAPK signaling pathways explain this process (Fig. S4A, F). Meanwhile, the over-reactive transcription factor HIF1α in CAFs of the hypermetabolic region promotes the transcription of downstream CXCL12,^29^ assisting the recruitment of Tregs and reshaping the TME for the purpose of immunosuppression.^30,31^

Hypoxia metabolism is a key factors that affect cellular expression reprogramming and TME reshaping by directly or indirectly regulating the expression of immune checkpoints and cytokine secretion.^26^ In the current study, the metabolism score from the bulk RNA sequencing data was significantly associated with the proportion of immune cells; it also proved to be valuable in the assessment of ICB responses. The high and low metabolic groups differed in the expression of key molecules, such as CXCL12, HIF1A, and SLC16A1.

## Conclusion

In the current study, the intratumorally metabolic heterogeneity of oral cancer was spatially investigated for the first time, and the relationship between hypermetabolic regions and local immune suppression was interpreted. The coordinate axis of cancer cells (lactate)/MCT1 (transformation from fibroblast to iCAF)/secretion of CXCL12 (promoting transcription by HIF1A)/Treg recruitment (CXCL12-ACKR3)/TGF-β1 secretion (tumor immune suppression microenvironment) was discovered by spatial transcriptomics and validated by immune fluorescence staining and bulk RNA-sequencing data of OSCC. However, clinical correlation analysis was limited by the sample size, and the annotation of CD4 + T cell lineage did not achieve the optimal resolution due to the limited features captured. Future research with more comprehensive data and validations using in vitro and in vivo assays is warranted.

## Materials and methods

### Sample collection

The study was approved by the Ethics Committee of the Ninth People’s Hospital, Shanghai Jiao Tong University School of Medicine (SH9H-2020-TK10-1). Fresh tumor and normal mucosa tissues were collected from patients with OSCC undergoing surgical resection in Shanghai Ninth People’s Hospital. All patients were untreated with chemo- or radiotherapy prior to surgery. All diagnoses were verified by an experienced histologist. Written informed consent was obtained from each patient. The clinical characteristics of the patients, including age, sex, stage, histological grade, and smoking status, are shown in Table 1.

**Table 1:** Clinical characters of three patients

Data Type	Patients ID	Age	Gender	Pathological T Stage	Pathological N Stage	Histological Grade	Smoking
Spatial	P1	85	Female	T1	N0	I	No
	P2	44	Male	T2	N0	II	No
	P3	41	Male	T2	N2	I	Yes
Single cell	P8	49	Female	T2	N1	I	No

### Sample preparation and data generation

For spatial transcriptome, the surgically resected tissues were immediately placed in an OCT-filled mold and snap-frozen in isopentane and liquid nitrogen. Cryosections were stored at −80 °C until use. Five to ten pieces of 5 µm-thick slices were taken, and RNA was extracted and immediately analyzed using the RNeasy Mini Kit (#74104; Qiagen, Hilden Germany). An RNA integrity number greater than seven was considered a qualified sample. The Visium Spatial Tissue Optimization slide was used to optimize the permeabilization conditions for the tumor and normal tissues, according to the 10× Visium spatial tissue optimization protocol. For cryosectioning, the samples were equilibrated to −20 °C. Blocks were trimmed to less than 6.5 mm × 6.5 mm before sectioning directly onto the Visium Spatial Tissue Optimization slide. The final optimized permeabilization time was 6–30 min for different tissue blocks. Once optimal conditions were established, three cryosections per patient were cut at a thickness of 10 μm onto spatial slides and immediately processed.

The 10× Visium spatial gene expression protocol was followed. The sectioned slides were stored at −20 °C for 30 min, whereas the sectioned slides for staining were incubated in hematoxylin (#BCCC7207, SIGMA) for 10 min, bluing buffer (#091824, Dako) for 2 min, and eosin (#17372-87-1, Merck) diluted 1:9 in Tris-base for 1 min. The slides were washed with RNAse- and DNAse-free water after each staining step. Thereafter, the slides were scanned at 40× magnification under a microscope (Leica DMI8 and CS2).

The slides were inserted into slide cassettes to separate tissue sections into individual reaction chambers. The permeabilization enzyme from the 10× Visium Gene Expression Kit (PN-1000184) was added to the tissue on the gene expression slide for the appropriate time according to the tissue optimization step. After incubation, the permeabilization enzyme was removed and the wells were washed with 0.1× SSC (#SLCF2892, SIGMA) before reverse transcription (RT) on the PCR instrument. After RT, the wells were washed with 0.1× SSC. Then, 75 μL 0.08 M KOH was added to each well for 5 min at 25 °C before adding the Second Strand Mix in a 10× Visium Gene expression Kit (PN-1000184) to the slide to generate the second strand. Thereafter, 0.08 mol/L KOH (diluted from stock) was added to each well for 10 min at 25 °C. qPCR was used to quantify the cDNA yield. Based on the Cq value, the double-stranded DNA was sequenced using PCR. Briefly, the library was prepared, followed by fragmentation, end repair, A-tailing, ligation, and index PCR. The libraries were sequenced on the Illumina NovaSeq platform and the resulting data were processed using SpaceRanger v1.1.0 (10× Genomics, Pleasanton, CA, USA) with manual alignment of fiducial markers and manual tissue identification.

For single cell transcriptome, tissue sample was temporarily stored in ice-cold storage buffer (RPMI 1640 + 0.04% BSA) before being washed twice with storage buffer and cut into approximately 0.5 mm^3^ pieces. These pieces were incubated in a fresh enzyme mixture at 37 °C for 30–60 min and filtered using a 40 μm cell strainer. After centrifugation (4 °C, 300 g for 5 min), an equal volume of 1X Red Blood Cell Lysis Buffer (MACS, 130-094-183) was added to the cell pellets and maintained at 4 °C for 10 min before centrifugation (4 °C, 300 g for 5 min). The cells were then washed and resuspended in RPMI 1640. cDNA libraries were constructed using the 10×Genomics Chromium Next GEM Single Cell 3ʹ Reagent Kits v3.1 (1000268) following the manufacturer’s instructions, and sequencing was performed on the Illumina Nova 6000 PE150 platform. The Cell Ranger software pipeline (version 3.1.0) provided by 10×Genomics was used to demultiplex cellular barcodes, map reads to the genome and transcriptome using the STAR aligner, and down-sample reads as required to generate normalized aggregate data across samples, producing a matrix of gene counts versus cells.

### Spatial transcriptomics data processing

The quality control of all 20 661 spatial spots is shown in Table S1. The gene-spot matrices generated after the ST data processing from the ST and Visium samples were analyzed with the ‘Seurat’ package v4.1.1^32^ in R v4.1.0. Normalization across spots was performed with the ‘SCTransform’ function. All data from the six samples were combined using the ‘FindIntegrationAnchors’ function. Dimensionality reduction and clustering were performed with principal component analysis (PCA) using the ‘RunPCA’ function. Integration with GSE103322 and cell labeling was performed using the ‘FindTransferAnchors’ function. DEG analysis was performed using the ‘FindAllMarkers’ function. Functional enrichment analysis including Gene Ontology (GO) and Kyoto Encyclopedia of Genes and Genomes (KEGG) analysis was performed using the ‘clusterProfiler’ package.^33^ Metabolism signature enrichment analysis for spots of ST data was performed using the scMetabolism algorithm,^10^ which required count data for calculation (the hypoxia gene sets are shown in the supplementary data). K-means was used to cluster groups according to the metabolic signatures in spots, and three clusters were generated.

### Single cell transcriptomics data processing

The UMI count matrix was preprocessed using the Seurat R package (version 4.1.2) to eliminate low-quality cells. A specific set of criteria was applied to filter cells, including: (1) removal of cells whose UMI/gene numbers were outside the mean value +/− 2-fold of standard deviations, based on the assumption of a Gaussian distribution for UMI/gene numbers for each cell, and (2) exclusion of cells with a percentage of mitochondrial RNA UMIs (i.e., the proportion of UMIs mapped to mitochondrial genes) greater than 10%. Following the application of these QC criteria, a total of 6628 single cells were retained for downstream analyses. Normalization of the counts was performed using the SCTransform function in Seurat.

### Cell lines and cell cultures

Cancer associated fibroblasts (CAFs) were isolated from tumor tissues of OSCC patients underwent radical surgery. The cells were cultured in Dulbecco’s modified Eagle’s medium (DMEM; Gibco-BRL, USA) supplemented with 10% fetal bovine serum (FBS; Gibco-BRL), penicillin (100 units per mL), and streptomycin (100 μg/mL) at 37 °C in a humidified 5% CO_2_ atmosphere.

### Multiple immunofluorescences staining and analysis

To avoid non-specific staining of antibodies from the same species, multiple immunofluorescence staining was performed using the TSA Plus kit based on tyramine signal amplification technics according to the manufacturer’s instructions. Spontaneous fluorescence was removed from paraffin sections using a tissue spontaneous fluorescence quencher immediately after antigen retrieval. All tissue slices were scanned by a Pannoramic Scanner using Pannoramic DESK, P-MIDI, and P250 (3D HISTECH, Hungary). Detailed information on commercial kits and antibodies can be found in the key resources table.

### TCGA bulk RNA-seq data acquisition and processing

Gene expression data, including read counts and fragments per kilobase of transcript per million mapped reads (FPKM), and the corresponding clinical information of patients with oral tongue SCC (OSCC, 128 cases) were downloaded from the HNSC projects of TCGA database (https://genome-cancer.ucsc.edu/). Patients diagnosed with OSCC and those with complete follow-up data were included. The FPKM data were transformed into transcripts per million (TPM) reads for further analyses. The metabolism signature enrichment analysis for each sample was calculated using the same method as for the ST data. The prediction of response to immune checkpoint blockade therapy was calculated using the online tool TIDE (http://tide.dfci.harvard.edu).^24^ Detailed prediction results are listed in Supplementary Materials.

### Analysis of immune infiltration

The analysis of the immune infiltration in TCGA OSCC was conducted using MCPcounter, and the single sample Gene Set Enrichment Analysis (ssGSEA) method was performed using the GSVA package (http://www.bioconductor.org/packages/release/bioc/html/GSVA.html) in R. Based on the signature genes reported in the literature ^23,34^ the relative enrichment score of all immune cells was calculated based on the gene expression profile deduced for each tumor sample.

### Cell-cell interactions

CellPhoneDB,^11^ a curated database of ligands, receptors, and their subunit interactions, was used to identify ligand–receptor interactions in the tumor samples of the three patients. Following identification of different cell types in our ST data, we followed the recommended protocol for preparation of input files and performed cell–cell interactions using the “*statistical_analysis*” function in CellPhoneDB v2.0. A functional understanding of cell–cell communication requires knowledge of the influence of these ligand–receptor interactions on target gene expression. NicheNet^12^ can predict which ligands influence the expression in another cell, the target genes affected by each ligand, and the signaling mediators that may be involved. Thus, to evaluate functional changes in receptor cells, NicheNet was chosen for the prediction of target genes in receptor cells. The visualization of the results from CellPhoneDB and NicheNet was performed using ‘ktplots’ and ‘ggplot2’ R packages.

### RNA extraction and RT-qPCR

Total RNA was extracted using TRIzol (Invitrogen, CA, United States). An equal amount of RNA was reverse- transcribed using the HiScript II Q RT Supermix and was quantified by qPCR using SYBR Green (Bimake). The primer sequences are shown in Supplementary Material.

### Statistical analysis and data visualization

All statistical analyses were performed using R v4.1.2. Gene expression between the two groups was compared using Wilcoxon rank-sum tests. Statistical significance was set at *P* < 0.05. The data were visualized using the ‘ggplot2’ and ‘ggsci’ packages.

### Supplementary information

Figure S1

Figure S2

Figure S3

Figure S4

Figure S5

Figure legends of supplementary figures

Multiple immunofluorescences antibodies

Primers

ST metabolism signature results

Figure 3 Cell data deconvolution results

Figure 3 SC data deconvolution results

Figure 6A cell_data_Fibroblast ligand GO KEGG

Figure 6A SC_data_Fibroblast ligand GO KEGG

Figure 9C cell_data Fibroblast Fibroblast targets Function

Figure 9C SC_data Fibroblast targets Function

Figure11 TIDE

Table S1

Table S2

Table S3

Table S4
