# Supplementary figures and images for: Spatial transcriptomics reveals that metabolic characteristics define the tumor immunosuppression microenvironment via iCAF transformation in oral squamous cell carcinoma
Source: Int J Oral Sci. 2024 Jan 30;16:9. doi: 10.1038/s41368-023-00267-8 (PMC10824761; doi:10.1038/s41368-023-00267-8)

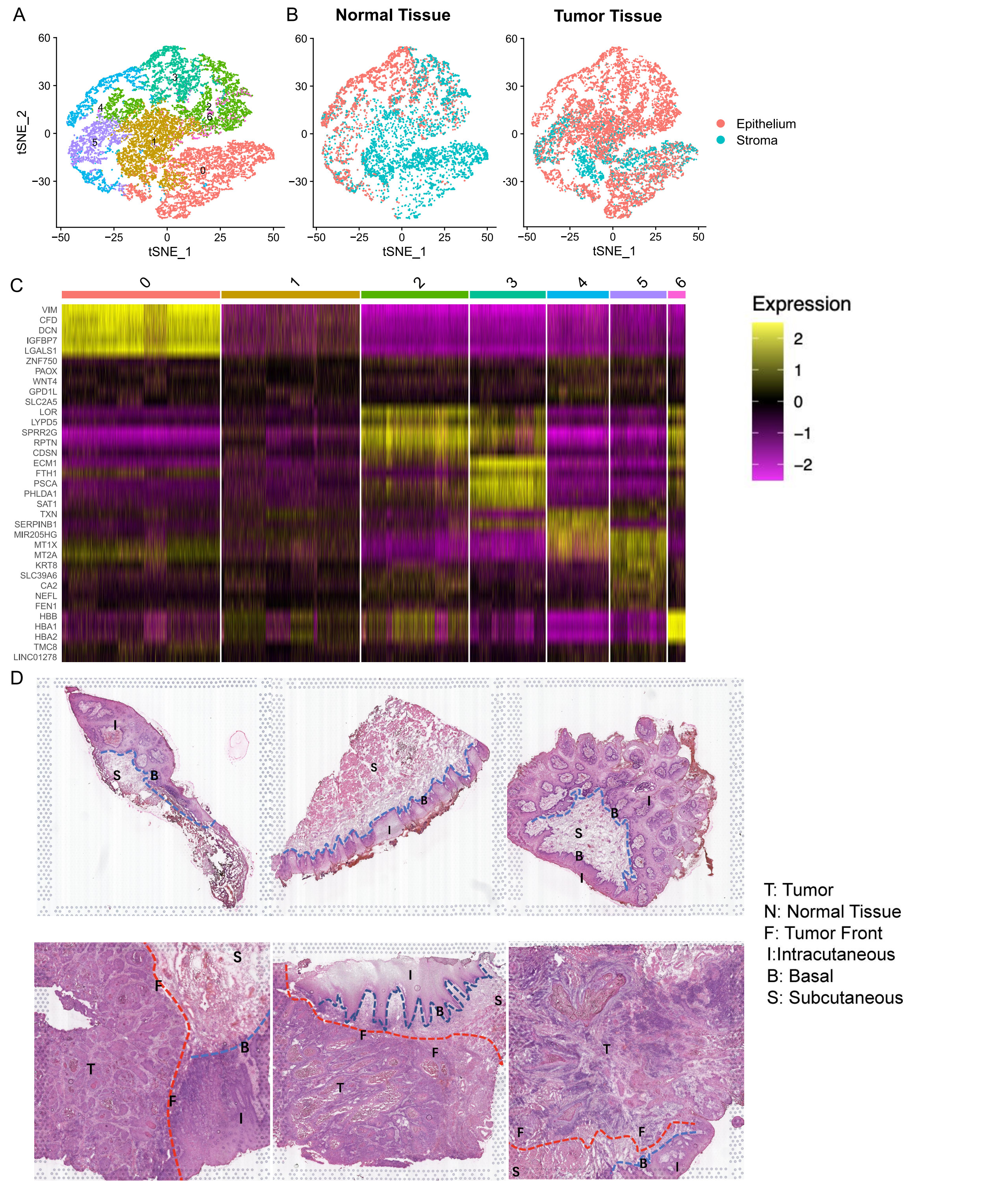

Supplement: Supplementary file 1 — Figure S1 [file 41368_2023_267_MOESM1_ESM.jpg]

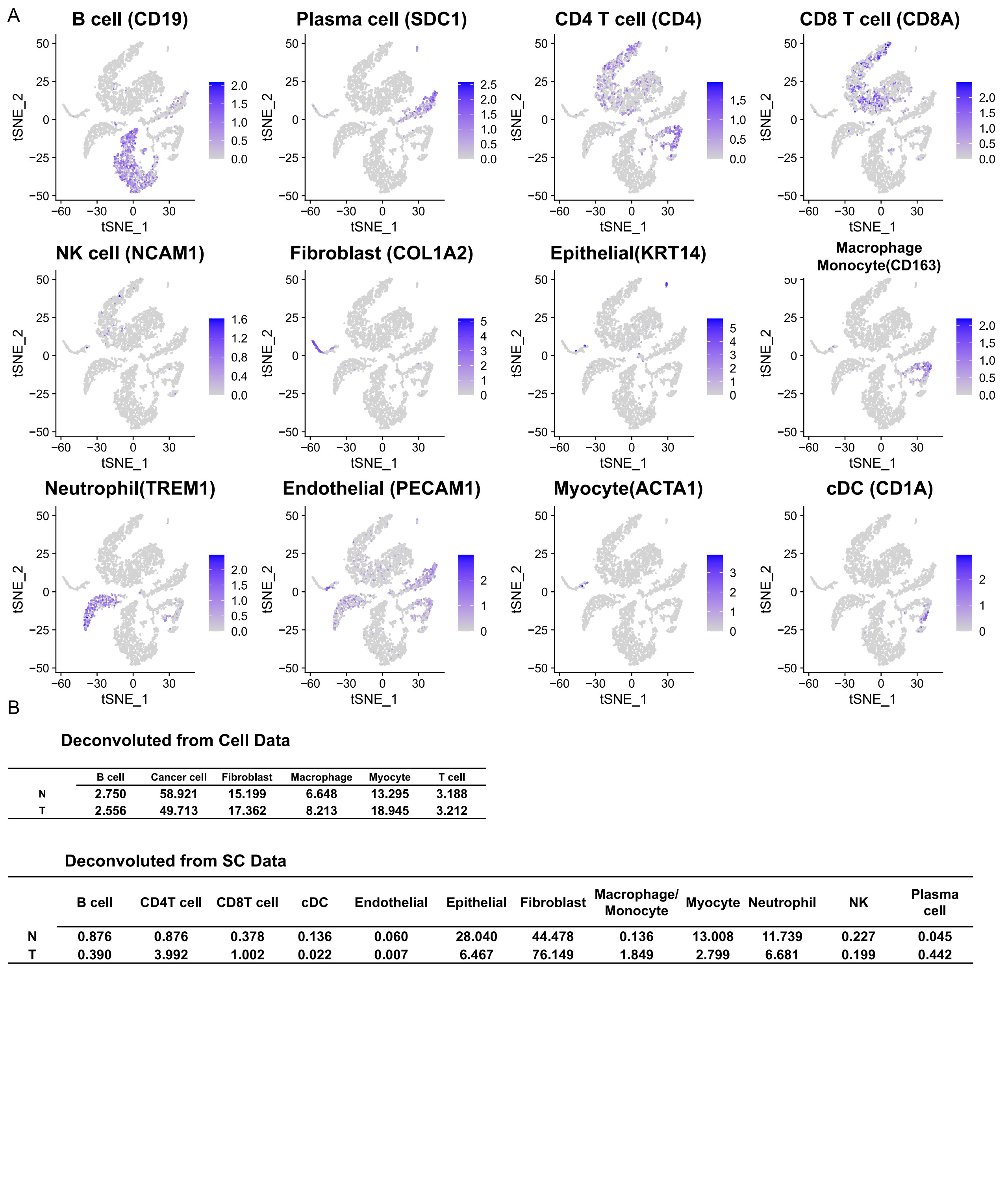

Supplement: Supplementary file 2 — Figure S2 [file 41368_2023_267_MOESM2_ESM.jpg]

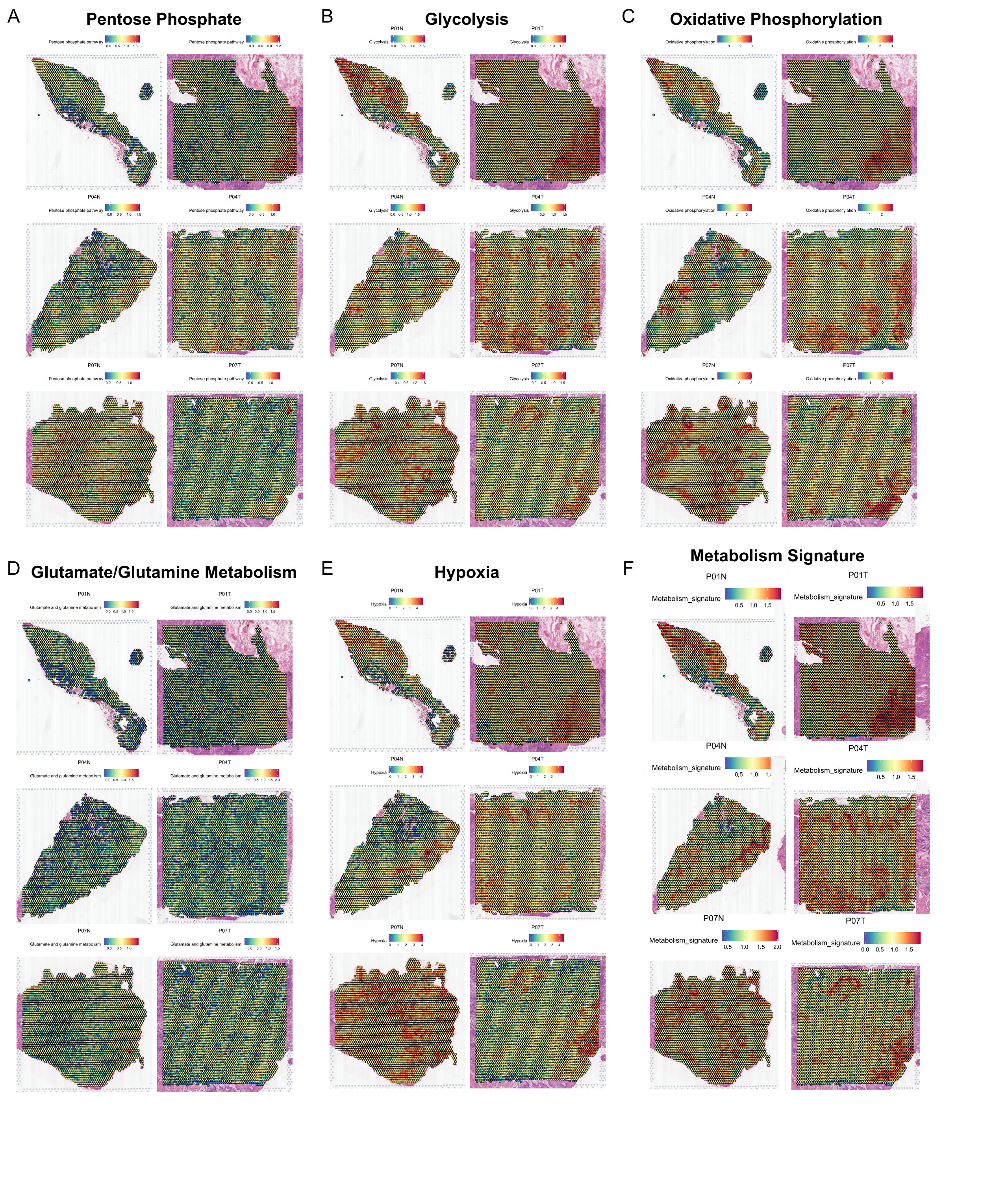

Supplement: Supplementary file 3 — Figure S3 [file 41368_2023_267_MOESM3_ESM.jpg]

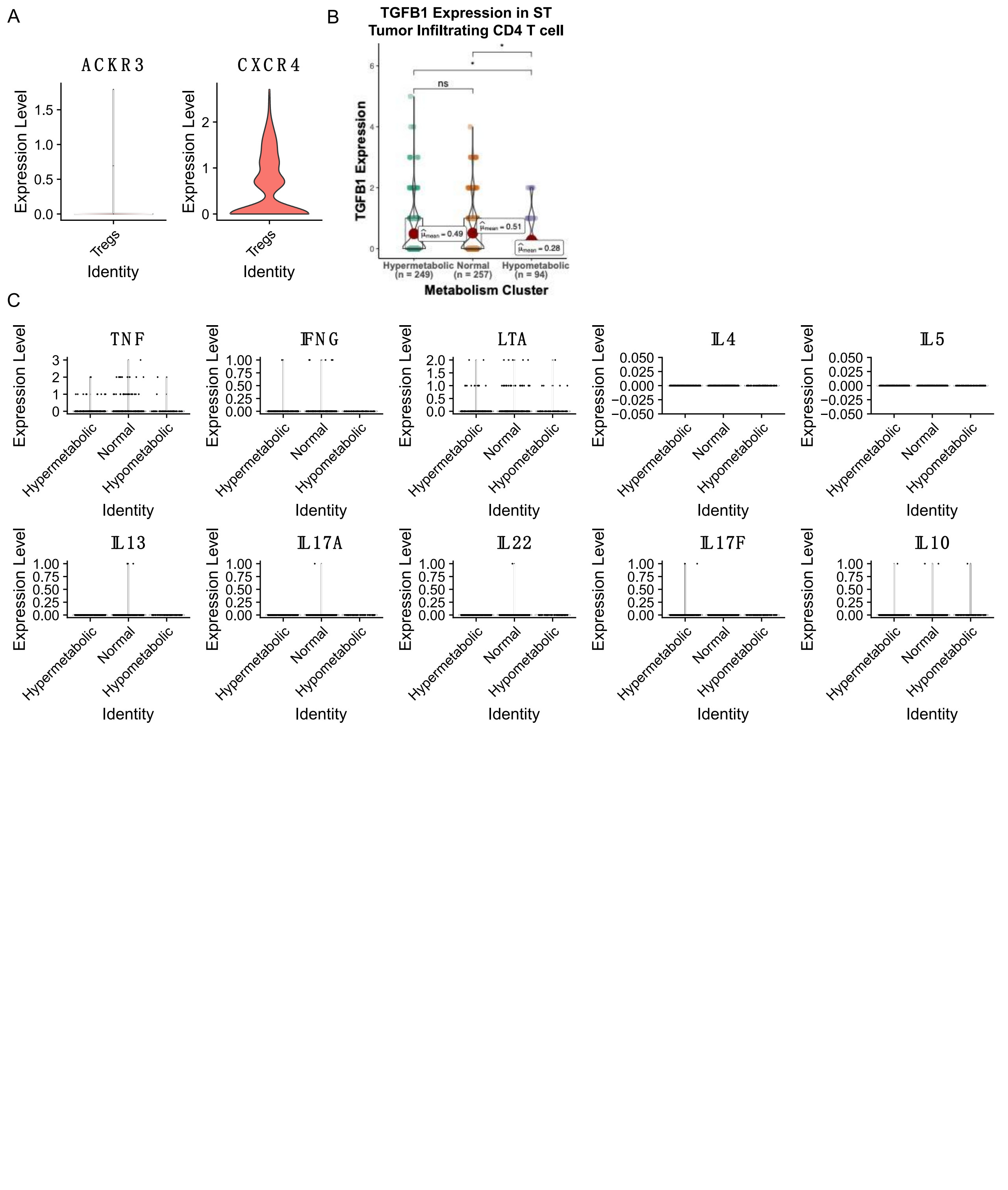

Supplement: Supplementary file 4 — Figure S4 [file 41368_2023_267_MOESM4_ESM.jpg]

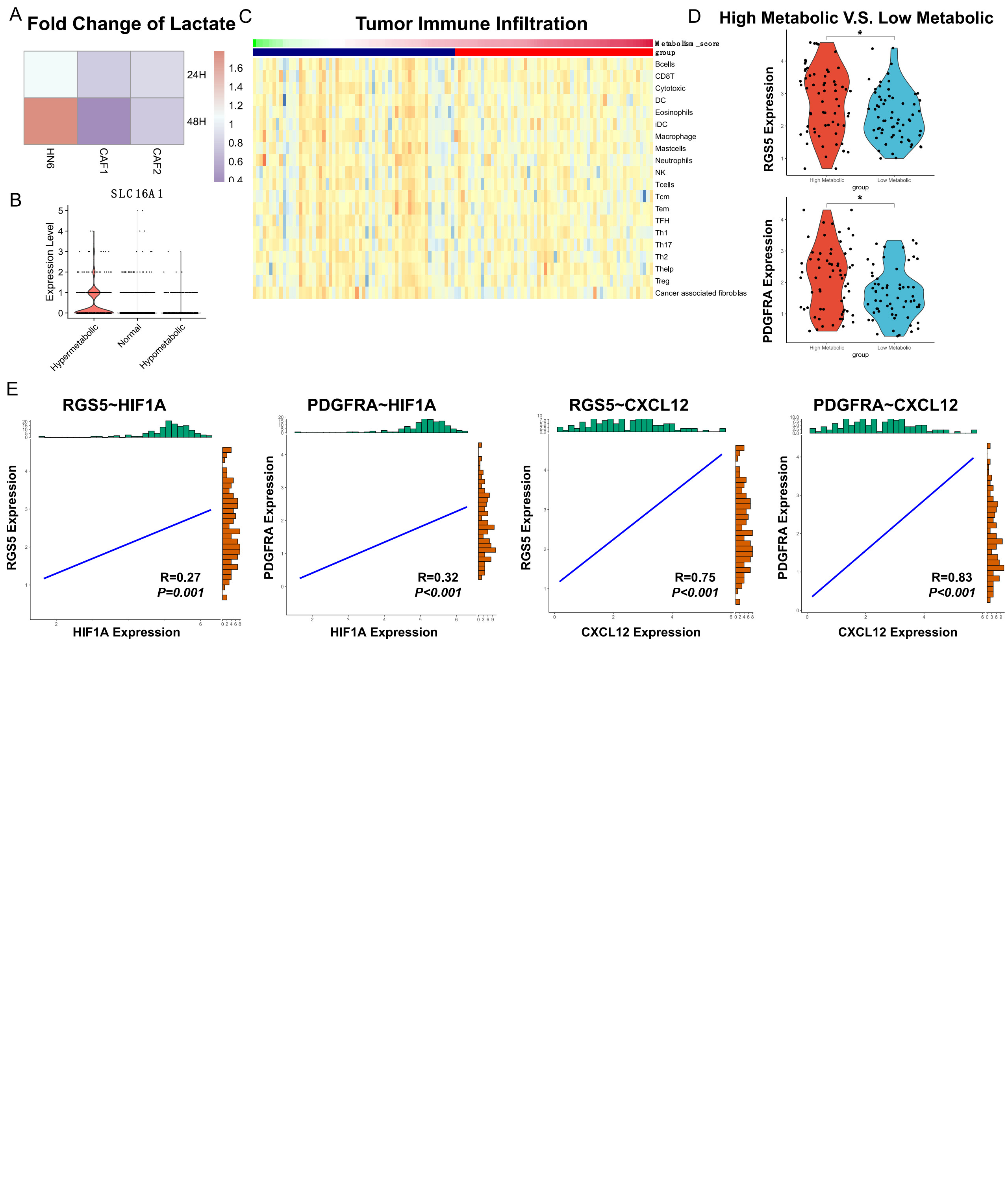

Supplement: Supplementary file 5 — Figure S5 [file 41368_2023_267_MOESM5_ESM.jpg]
