# Supplementary material for: Spatial transcriptomics reveals that metabolic characteristics define the tumor immunosuppression microenvironment via iCAF transformation in oral squamous cell carcinoma
Source: Int J Oral Sci. 2024 Jan 30;16:9. doi: 10.1038/s41368-023-00267-8 (PMC10824761; doi:10.1038/s41368-023-00267-8)
Supplement: Supplementary file 6 — Figure legends of supplementary figures [file 41368_2023_267_MOESM6_ESM.pdf]

Figure S1.

(A)The t-Distributed Stochastic Neighbor Embedding (t-SNE) plot shows the spatial distribution of ST-seq spots recovered from six tissue samples (three adjacent normal tissues and three tumor tissues) obtained from three patients. (B)The t-SNE plot shows the distribution of ST-seq spots within different tissue regions. (C) Heatmap showing the marker genes of unsupervised clusters of all tissues spots. (D) The annotations of different tissues regions by the pathologist.

Figure S2.

(A) Expression distribution feature plot of markers for various cell types in SC data. (B) Detailed percentage of different cell types of Cell data and SC data in Normal and tumor samples.

Figure S3.

(A) to (F) Projection of 5 different metabolism scores, including glycolysis, pentose phosphate, oxidative phosphorylation, glutamate/glutamine metabolism and hypoxia, and combined metabolism signature score on the 6 tissue slides.

Figure S4.

(A) Expression of ACKR3 and CXCR4, which were receptors of CXCL12, in Tregs. ACKR3 rarely had expression in Tregs. (B) TGFB1 expression was highly upregulated in hypermetabolic regions among tumor infiltrating CD4 T cells in ST data. (C)The expression of cytokines secreted by different types of T cells in different metabolic regions, including TNF-α (TNF), interferon gamma (IFG), LTA, IL4, IL5, IL13, IL17A, IL17F, IL22 and IL10. All of these cytokines expression had no changes in different metabolic regions.

Figure S5.

(A) Expression fold change of lactate concentration in HN6 cell line and 2 CAF cell lines in High/Low glucose culture medium. (B) SLC16A1 expression among different metabolic regions in fibroblasts. (C) Heatmap showing the tumor immune infiltration using ssGSEA and

MCPCounter. (D) The expression of iCAF markers, RGS5 and PDGFRA, was higher in high metabolic samples than in low metabolic samples in TSCC. (E) Correlation scatter plot indicated the high positive correlation of RGS5 to HIF1A, PDGFRA to HIF1A, RGS5 infiltration to CXCL12, and PDGFRA to CXCL12 in TSCC samples
