# Supplementary material for: Spatial transcriptomics reveals that metabolic characteristics define the tumor immunosuppression microenvironment via iCAF transformation in oral squamous cell carcinoma
Source: Int J Oral Sci. 2024 Jan 30;16:9. doi: 10.1038/s41368-023-00267-8 (PMC10824761; doi:10.1038/s41368-023-00267-8)
Supplement: Supplementary file 7 — Multiple immunofluorescences antibodies [file 41368_2023_267_MOESM7_ESM.docx]

Multiple immunofluorescences staining antibodies

| Reagents | Identifier | Source |
| --- | --- | --- |
| Anti-PDGFRA | GB111342 | Servicebio |
| Anti-IL-6 | GB11117 | Servicebio |
| Anti-FOXP3 | GB112325 | Servicebio |
| Anti-FAP | GB11096 | Servicebio |
| Anti-aSMA | GB13044 | Servicebio |
| Anti-LDHA | GB11342 | Servicebio |
| Anti-HIF-1A | GB114936 | Servicebio |
| Anti-CXCL12 | GB11624 | Servicebio |
| CY3-labeled goat anti-rabbit IgG | GB21303 | Servicebio |
| CY3-labeled goat anti-mouse IgG | GB21301 | Servicebio |
| CY3-labeled goat anti-rat IgG | GB21302 | Servicebio |
| CY3-labeled donkey anti-goat IgG | GB21404 | Servicebio |
| CY3-labeled donkey anti-mouse IgG | GB21401 | Servicebio |
| CY3-labeled donkey anti-rabbit IgG | GB21403 | Servicebio |
| Alexa Fluor 488-labeled goat anti-rabbit IgG | GB25303 | Servicebio |
| Alexa Fluor 488-labeled goat anti-mouse IgG | GB25301 | Servicebio |
| CY5-labeled goat anti-mouse IgG | GB27301 | Servicebio |
| CY5-labeled goat anti-rabbit IgG | GB27303 | Servicebio |
| HRP-labeled rabbit anti-goat IgG | GB23204 | Servicebio |
| HRP-labeled goat anti-mouse IgG | GB23301 | Servicebio |
| HRP-labeled goat anti-rat IgG | GB23302 | Servicebio |
| HRP-labeled goat anti-rabbit IgG | GB23303 | Servicebio |
| HRP-labeled donkey anti-goat IgG | GB23404 | Servicebio |
| FITC-labeled donkey anti-goat IgG | GB22404 | Servicebio |
| FITC-labeled goat anti-rat IgG | GB22302 | Servicebio |
| FITC-labeled donkey anti-rabbit IgG | GB22403 | Servicebio |
| FITC-labeled donkey anti-mouse IgG | GB22401 | Servicebio |
| Alexa Fluor 594-labeled goat anti-rabbit IgG | 111-585-003 | Jackson |
| Alexa Fluor 594-labeled goat anti-mouse IgG | 115-585-003 | Jackson |
| CY3-Tyramide | G1223 | Servicebio |
| iF488-Tyramide | G1231 | Servicebio |
| iF647-Tyramide | G1232 | Servicebio |
| FITC-Tyramide | G1222 | Servicebio |
