# Supplementary material for: Spatial transcriptomics reveals that metabolic characteristics define the tumor immunosuppression microenvironment via iCAF transformation in oral squamous cell carcinoma
Source: Int J Oral Sci. 2024 Jan 30;16:9. doi: 10.1038/s41368-023-00267-8 (PMC10824761; doi:10.1038/s41368-023-00267-8)
Supplement: Supplementary file 8 — Primers [file 41368_2023_267_MOESM8_ESM.docx]

Primers of RT-qPCR

| Genes | Forward | Reverse |
| --- | --- | --- |
| HIF1A | GAACGTCGAAAAGAAAAGTCTCG | CCTTATCAAGATGCGAACTCACA |
| RGS5 | CCAGAAGCCAGACTCTGCTGTTGAC | CATAGATTTGCTTTGCCTTCTCTG |
| CXCL12 | ATTCTCAACACTCCAAACTGTGC | ACTTTAGCTTCGGGTCAATGC |
| PDGFRA | TGGCAGTACCCCATGTCTGAA | CCAAGACCGTCACAAAAAGGC |
| Actin | CATGTACGTTGCTATCCAGGC | CTCCTTAATGTCACGCACGAT |
