# Supplementary material for: Spatial transcriptomics reveals that metabolic characteristics define the tumor immunosuppression microenvironment via iCAF transformation in oral squamous cell carcinoma
Source: Int J Oral Sci. 2024 Jan 30;16:9. doi: 10.1038/s41368-023-00267-8 (PMC10824761; doi:10.1038/s41368-023-00267-8)
Supplement: Supplementary file 17 — Table S1 [file 41368_2023_267_MOESM17_ESM.pdf]

Tabele S1. Quality control of each sample

Metrics	01 Normal	04 Normal	07 Normal	01 Tumor	04 Tumor	07 Tumor
Number of Spots Under Tissue	1500	2325	3043	4272	4679	4842
Median Genes per Spot	1052	1385	3206	2348	2848	2734
Number of Reads	66780027	130294536	224081848	236180722	255386083	241659545
Valid Barcodes	96.9%	97.1%	96.9%	96.5%	96.6%	96.4%
Valid UMIs	100%	99.9%	100%	99.9%	99.9%	99.9%
Mean Reads per Spot	44520	56041	73638	55286	54581	49909
Sequencing Saturation	65.6%	74.6%	63.5%	75.4%	73.5%	73.3%
Q30 Bases in Barcode	95.7%	96.7%	96%	96.1%	95.7%	95.7%
Q30 Bases in RNA Read	90.8%	92.1%	90.4%	89.6%	88%	89.2%
Q30 Bases in UMI	94.5%	93.7%	95.4%	95.5%	94.8%	94.5%
Reads Mapped to Genome	92.6%	87.6%	94.1%	89.4%	80.6%	87.7%
Reads Mapped Confidently to Genome	82.8%	76.9%	90.1%	80.5%	75.4%	82.7%
Reads Mapped Confidently to Intergenic Regions	5.7%	5.8%	4.3%	2.9%	3.1%	1.8%
Reads Mapped Confidently to Intronic Regions	4%	5.6%	2.3%	5.1%	4%	3.1%
Reads Mapped Confidently to Exonic Regions	73.1%	65.5%	83.5%	72.5%	68.3%	77.7%

Metrics	01 Normal	04 Normal	07 Normal	01 Tumor	04 Tumor	07 Tumor
Reads Mapped Confidently to Transcriptome	71.2%	63.4%	81.5%	70.3%	66.3%	75.2%
Reads Mapped Antisense to Gene	0.5%	0.5%	0.5%	0.8%	0.6%	1%
Fraction Reads in Spots Under Tissue	36.8%	86.7%	78.7%	91.7%	98.4%	97.1%
Total Genes Detected	19010	22260	22434	23392	24635	24273
Median UMI Counts per Spot	2606	3095	13420	5338	7126	7485
Fraction of Spots Under Tissue	30%	46.6%	61%	85.6%	93.7%	97%
