# Supplementary material for: Spatial transcriptomics reveals that metabolic characteristics define the tumor immunosuppression microenvironment via iCAF transformation in oral squamous cell carcinoma
Source: Int J Oral Sci. 2024 Jan 30;16:9. doi: 10.1038/s41368-023-00267-8 (PMC10824761; doi:10.1038/s41368-023-00267-8)
Supplement: Supplementary file 18 — Table S2 [file 41368_2023_267_MOESM18_ESM.pdf]

Tabele S2. Predicted Ligands from Fibroblast by Nichenet

Deconvolution Data	Ligands Predicted
Cell data	DSC3
	LAMA2
	APP
	IGF1
	IL15
	ADAM17
	APOE
	CXCL12
	CCL2
	SFRP2
	LTB
	CADM1
SC data	DSC3
	APP
	JAG1
	HMGB2
	CALR
	NRG1
	TGFB1
	APOE
	IGF1
	LAMA2
	LAMB2
	SFRP2
	MMP13
	CXCL12
	COL5A3
	ITGAL
	DLL1
	TGFB3
