# Supplementary material for: Spatial transcriptomics reveals that metabolic characteristics define the tumor immunosuppression microenvironment via iCAF transformation in oral squamous cell carcinoma
Source: Int J Oral Sci. 2024 Jan 30;16:9. doi: 10.1038/s41368-023-00267-8 (PMC10824761; doi:10.1038/s41368-023-00267-8)
Supplement: Supplementary file 19 — Table S3 [file 41368_2023_267_MOESM19_ESM.pdf]

Table S3 Ligands-Targets genes matrix in T cells of Cell Data

Targets genes	Overlapped 7 Ligands						
	DSC3	LAMA2	APP	IGF1	APOE	CXCL12	SFRP2
AR	0.00145802	0	0.00426135	0.00143412	0	0	0
EHF	0	0	0	0	0	0	0.0014834
GATA3	0.00153533	0.0039342	0	0	0	0	0.00175145
SOX17	0.00171058	0	0	0	0	0	0.00149118
ACACB	0	0.00593034	0	0	0	0	0
CARD18	0	0	0.00956467	0	0	0	0
FYB1	0	0	0.01488517	0	0	0	0
SBK1	0	0	0.00957867	0	0	0	0
CHRNA10	0	0	0	0.00816465	0	0	0
CLEC5A	0	0	0.00638129	0.0066239	0	0	0
COL17A1	0	0	0	0.00703821	0	0	0
LAMC3	0	0	0	0.00687119	0	0	0
RLN2	0	0	0	0.00808355	0	0	0
SLC7A5	0	0.004301	0	0.00613325	0	0	0
TRBC2	0	0	0	0.02851115	0	0	0
TCN1	0	0	0.00719823	0	0	0	0
APP	0	0	0.00390611	0.00219514	0	0	0
CAT	0	0	0.00393927	0	0	0	0
CCL19	0	0	0	0	0.00646086	0	0
CXCR4	0	0	0	0	0.0038683	0.00514401	0.00175276
HLA.E	0	0	0.00400583	0	0	0	0
IL10RA	0	0	0.00418046	0	0	0	0
KRT8	0	0.00449636	0	0	0	0	0
SLC2A1	0	0	0	0.00276536	0	0	0
VEGFC	0	0	0	0.00246503	0	0	0
ADH7	0	0	0	0	0.00632217	0	0
C1QB	0	0	0	0	0.0056501	0	0
CCL4	0	0	0.00208857	0	0.00450175	0	0
SELPLG	0	0	0	0	0.00459194	0	0
ITK	0	0	0	0	0	0.00268502	0
MT2A	0	0	0	0	0	0.00431805	0
PTPRC	0	0	0.00466007	0	0	0	0
AGER	0	0	0.00445822	0	0	0	0.00158969
GALNT17	0	0	0	0	0	0	0.00225546
GSC	0	0	0	0	0	0	0.00195753
OSR1	0	0	0	0	0	0	0.00174303
PDE10A	0	0	0	0	0	0	0.00197943
GAPDH	0	0	0	0.00142386	0	0	0
LAPTM5	0	0	0.00437603	0	0	0	0
LCP2	0	0	0.00476086	0	0	0	0

OLFM1	0	0	0.00494824	0	0	0	0
