# Supplementary material for: Spatial transcriptomics reveals that metabolic characteristics define the tumor immunosuppression microenvironment via iCAF transformation in oral squamous cell carcinoma
Source: Int J Oral Sci. 2024 Jan 30;16:9. doi: 10.1038/s41368-023-00267-8 (PMC10824761; doi:10.1038/s41368-023-00267-8)
Supplement: Supplementary file 20 — Table S4 [file 41368_2023_267_MOESM20_ESM.pdf]

Table S4 Ligands-Targets genes matrix in CD4 T cells of SC Data

Targets genes	Overlapped 7 Ligands						
	DSC3	LAMA2	APP	IGF1	APOE	CXCL12	SFRP2
CCND1	0.00169209	0	0.00346708	0.00477044	0.00147858	0.00156572	0.00208892
EHF	0.00140847	0	0	0	0	0	0.0014834
FOXC1	0.00161649	0	0	0	0	0	0.00144848
GATA2	0.00144275	0	0	0.00240165	0	0	0
MYC	0.00193515	0	0.00145071	0.00261188	0	0	0.00212002
C7orf57	0	0	0.01168328	0	0	0	0
CARD18	0	0	0.00956467	0	0	0	0
LCE2C	0	0	0.01382793	0	0	0	0
PLPP7	0	0	0.01719258	0	0	0	0
CDK1	0	0	0.00392535	0.00423099	0	0	0
CITED2	0	0	0	0.00447625	0	0	0.00167039
HSP90AB1	0	0	0	0.00176654	0	0	0
PCNA	0	0	0.00385697	0.00223282	0	0	0
PTMA	0	0	0.00137739	0.00168649	0	0	0.00146256
FOS	0	0	0.00536461	0.00239193	0	0.00141949	0.00156203
NFE2L2	0	0	0	0.00153136	0.00415633	0	0.00160846
NOS3	0	0	0	0.00142362	0.00460863	0	0
RANBP1	0	0.00422711	0	0	0	0	0
TUBB	0	0	0	0.00141274	0	0	0
ACTA2	0	0	0	0.0039884	0.00377844	0	0
JAG1	0	0	0	0	0	0	0.00178708
IL1A	0	0.00437515	0	0	0.00440691	0	0
LCN2	0	0	0	0	0.00403772	0	0
PTGR1	0	0	0	0	0.004503	0	0
SLC5A1	0	0	0	0	0.00533441	0	0
COL17A1	0	0	0	0.00703821	0	0	0
IL36RN	0	0	0	0.00658887	0	0	0
KRT2	0	0	0	0.00690138	0	0	0
RLN2	0	0	0	0.00808355	0	0	0
SPRR2B	0	0	0	0.0076857	0	0	0
ATP12A	0	0.00610316	0	0	0	0	0
FGFBP1	0	0.00555862	0	0	0	0	0
GCNT3	0	0.00585375	0.00566219	0	0	0	0
NRIP2	0	0.00611283	0	0	0	0	0
PLA2G2F	0	0.00676045	0	0	0	0	0
PRR9	0	0.00670736	0	0	0	0	0
OSR1	0	0	0	0	0	0	0.00174303
RASAL2	0	0	0	0	0	0	0.00160473
SEMA6D	0	0	0	0	0	0	0.00170239
MAGI1	0	0	0	0	0	0	0.00150966

CEACAM1	0	0	0	0	0	0.00200304	0
MT2A	0	0	0	0	0	0.00431805	0
DUOX2	0	0	0.00255253	0	0	0	0
SLC26A9	0	0	0.00573181	0	0	0	0
GAPDH	0	0	0	0.00142386	0	0	0
